# Typologies of Family Functioning and 24-h Movement Behaviors

**DOI:** 10.3390/ijerph18020699

**Published:** 2021-01-15

**Authors:** Michelle D. Guerrero, Joel D. Barnes, Mark S. Tremblay, Laura Pulkki-Råback

**Affiliations:** 1 Healthy Active Living and Obesity Research Group, Children’s Hospital of Eastern Ontario Research Institute, Ottawa, ON K1H 8L1, Canada; mtremblay@cheo.on.ca; 2 College of Kinesiology, University of Saskatchewan, Saskatoon, SK S7N 5B2, Canada; j@barnzilla.ca; 3 Department of Pediatrics, University of Ottawa, Ottawa, ON K1N 6N5, Canada; 4 Department of Psychology and Logopedics, Faculty of Medicine, University of Helsinki, 00100 Helsinki, Finland; laura.pulkki-raback@helsinki.fi; 5 Department of Clinical Medicine, Division of Child Psychiatry, University of Turku, 20014 Turku, Finland

**Keywords:** latent profile analysis, physical activity, sleep, screen time, family environment, children

## Abstract

Research on the importance of the family environment on children’s health behaviors is ubiquitous, yet critical gaps in the literature exist. Many studies have focused on one family characteristic and have relied on variable-centered approaches as opposed to person-centered approaches (e.g., latent profile analysis). The purpose of the current study was to use latent profile analysis to identify family typologies characterized by parental acceptance, parental monitoring, and family conflict, and to examine whether such typologies are associated with the number of movement behavior recommendations (i.e., physical activity, screen time, and sleep) met by children. Data for this cross-sectional observational study were part of the baseline data from the Adolescent Brain Cognitive Development (ABCD) study. Data were collected across 21 study sites in the United States. Participants included 10,712 children (female = 5143, males = 5578) aged 9 and 10 years (M = 9.91, SD = 0.62). Results showed that children were meaningfully classified into one of five family typologies. Children from families with *high acceptance, medium monitoring,* and *medium conflict* (P2; OR = 0.54; 95% CI, 0.39–0.76); *high acceptance*, *medium monitoring*, and *high conflict* (P3; OR = 0.28; 95% CI, 0.20, 0.40); *low acceptance*, *low monitoring*, and *medium conflict* (P4; OR = 0.24; 95% CI, 0.16, 0.36); and *medium acceptance*, *low monitoring*, and *high conflict* (P5; OR = 0.19; 95% CI, 0.12–0.29) were less likely to meet all three movement behavior recommendations compared to children from families with *high acceptance*, *high monitoring*, and *low conflict* (P1). These findings highlight the importance of the family environment for promoting healthy movement behaviors among children.

## 1. Introduction

The *Canadian 24-h Movement Guidelines* recommend that children and youth (5–13 years) accumulate a minimum of 60 min of moderate-to-vigorous physical activity per day, engage in no more than 2 h of screen time per day, and obtain 9–11 h of sleep per night [1]. Meeting these recommendations is linked to favorable body composition [2], lower cardiometabolic risk scores [3], favorable psychological well-being [4], higher global cognition scores [5], lower impulsivity scores [6], and lower depressive symptoms [7]. Many factors are implicated in the development and maintenance of these movement behaviors. One factor that plays a powerful role in shaping children’s health behaviors is the family environment [8,9].

Parenting practices generally refer to the specific acts of parents when attempting to socialize their children and can include parental acceptance (e.g., affection, approval, warmth, and support) [10] and monitoring (e.g., knowing where and with whom the child spends her time). These parenting practices safeguard children from risky health behaviors, promote positive health outcomes [11], and play a crucial role in children’s movement behaviors according to a recent consensus statement [12]. Numerous studies show positive associations between parental encouragement and support and children’s levels of physical activity [9,13]. The relationship between parental acceptance and screen time remains largely unknown, though it is likely complex given the research on parenting styles and screen time; authoritarian (low acceptance, high demand) and permissive (high acceptance, low demand) parenting styles are linked with greater screen time among 5–10-year-olds [14]. Moreover, parental monitoring of children’s sleep, physical activity, and media use is associated with longer sleep duration [15], greater physical activity [16], and less screen viewing among children [17], respectively. A family environment that lacks parental acceptance and supervision can lead to problems in several aspects of children’s health, such as sleep problems due to worry [18], increased time spent being sedentary, and increased reliance on screen devices.

In contrast to protective family factors, family conflict is considered a risk factor that may hinder children’s ability to meet the 24-h movement behaviors. Family conflict refers to openly expressed anger, aggression, and disagreement among family members [19], and it is believed to interfere with parenting ability [20]. Empirical evidence supports the notion that parents in high-conflict families may be unable to monitor their children’s physical activity, media use, and sleep habits. Family conflict is frequently linked with sleep disruptions and poor sleep quality in children [21,22]. In fact, family conflict during childhood (7–15 years) predicts insomnia later in life [23]. Marital conflict, in particular, has been shown to negatively impact children’s sleep as characterized by sleep onset latency, frequent awakenings, reduced sleep duration, and increased nightmares [24,25]. Associations between family conflict and media use suggest that higher family tension is linked with greater television viewing among children [26], and that children from high-conflict families (vs. those in less conflictual families) watch more violent electronic media [27].

Research on the importance of the family environment on children’s health behaviors is ubiquitous [12]. However, critical gaps exist in the literature. Many studies have generally focused on only one family characteristic or factor and its relationship with different health outcomes. Where multiple family characteristics are examined, researchers have often adopted “variable-centered” approaches, which assume that participants are drawn from a single, homogeneous population. Alternatively, “person-centered” approaches—such as latent profile analysis (LPA)—allow researchers to identify homogeneous groups, typologies, or profiles of participants characterized by differences on variables of interest (e.g., family environment). To date, no studies have examined different family environment characteristics and how they relate to children’s movement behaviors. Therefore, the purpose of the current study was to use LPA to identify typologies of families characterized by parental acceptance, parental monitoring, and family conflict, and to examine whether such family typologies are associated with the number of movement behavior recommendations met by children.

## 2. Materials and Methods

### 2.1. Study Design

These data (*N* = 11,875) are part of the baseline cross-sectional dataset from the Adolescent Brain Cognitive Development (ABCD) study, a broadly representative sample of 9 to 10-year-old children recruited from 21 research sites across the United States. A broad aim of the ABCD study is to track brain development of children for 10 years to understand how different factors (e.g., environmental, behavioral, biological) impact or alter developmental trajectories. The ABCD study is the largest long-term study of brain development and child health in the United States. Children and their parents/guardians were recruited by probability sampling of elementary schools within the catchment areas of the 21 data acquisition sites. Participants with missing data were removed from the data set. The final sample size was 10,712 participants. Ethics approval was obtained from all relevant institutional research ethics boards. Signed, informed consent and assent were obtained from parents/guardians and participating children, respectively.

### 2.2. Exposures

Parental acceptance, parental monitoring, and family conflict were used in the LPA analyses to create family profiles/typologies. Children’s perceptions of parental warmth, acceptance, and responsiveness were assessed using the acceptance subscale (5 items) of the Child Report of Behavior Inventory [28]. Items assess the extent to which children perceive their caregiver as warm or accepting (e.g., “Makes me feel better after talking over my worries with him/her”; “Smiles at me very often”). Items are rated on a three-point scale, ranging from 1 (*not like him/her*) to 3 (*a lot like him/her*). Children reported on their primary caregiver (usually the mother), who is also participating in the ABCD study. Parental monitoring was assessed using a scale (5 items) developed by the ACBD leadership group that assesses children’s perceptions of how often their parents: know where they are (“How often do your parents know where you are?”); who they are with (e.g., “How often do your parents know who you are with when you are not at home and away from home?”); communicate with them (“If you are at home and your parents or guardians are not, how often do you know how to get in touch with them?”); and know of their upcoming plans, and family dinner frequency. Family conflict was assessed using the conflict subscale (9 items) of the Moos Family Environment Scale [19]. Items assess the amount of openly expressed anger and conflict among family members using a true or false response scale. For each measure, items were summed, whereby higher scores reflect more acceptance, greater parental monitoring, and more conflict. Internal consistencies for the acceptance, monitoring, and conflict scales were acceptable (ω = 0.86, ω = 0.62, and ω = 0.83, respectively).

### 2.3. Outcomes

The movement behaviors served as the outcome variables. Physical activity was assessed using one item from the Youth Risk Behavior Survey [29], wherein children reported the number of days per week they were physically active for at least 60 min. Screen time was assessed using the child-reported Youth Screen Time Survey (12 items), which was developed by Barch et al. [30]. Items assess how much time children spend on different types of media (e.g., watching shows or movies, texting) on both a typical weekday and weekend day. Daily recreational screen time was calculated by taking a weighted average of the weekday and weekend screen time items: (sum of weekday screen time in decimal hours × 5) + (sum of weekend day screen time in decimal hours × 2)/7. Sleep was assessed using one item from the Sleep Disturbance Scale for Children [31]. Parents reported the number of hours of sleep their child obtained on most nights. For each movement behavior, children were coded as either 1 (meeting the guideline) or 0 (not meeting the guideline) and were then used to create three separate dichotomous variables: (1) 0 recommendations met vs. ≥1 recommendation met; (2) 0 recommendations met vs. ≥2 recommendations met; (3) 0 recommendations met vs. 3 recommendations met.

### 2.4. Statistical Analyses

Statistical analyses were conducted using a multiphase approach. First, we used LPA to determine whether participants could be classified into meaningful profiles based on the three dimensions of family environment (i.e., acceptance, monitoring, and conflict). Scores on acceptance, monitoring, and conflict were standardized and used in the LPA, which was conducted in R using the mclust package [32]. We used an iterative process to identify the optimal number of profiles whereby we started with a 2-profile solution and sequentially added profiles. Various criteria were used to identify the best fitting model, including the Bayesian Information Criterion (BIC), Consistent Akaike’s Information Criterion (CAIC), the Sample-Adjusted Bayesian Information Criterion (SABIC), and the Bootstrap Likelihood Ratio Test (BLRT). Lower BIC, CAIC, and SABIC values indicate better model fit, whereas the *p*-value generated for the BLRT indicates whether the solution with more classes or fewer classes fits better. Classification accuracy was evaluated using entropy, whereby values close to 1 indicate improved classification precision. Once the best-fitting profile was identified, multilevel logistic regressions were used to examine the association between family profiles and meeting the 24-h guidelines. All models were adjusted for age, sex, ethnicity, and family income and included study site as the random intercept.

## 3. Results

### 3.1. Latent Profiles 

**Figure 1:**
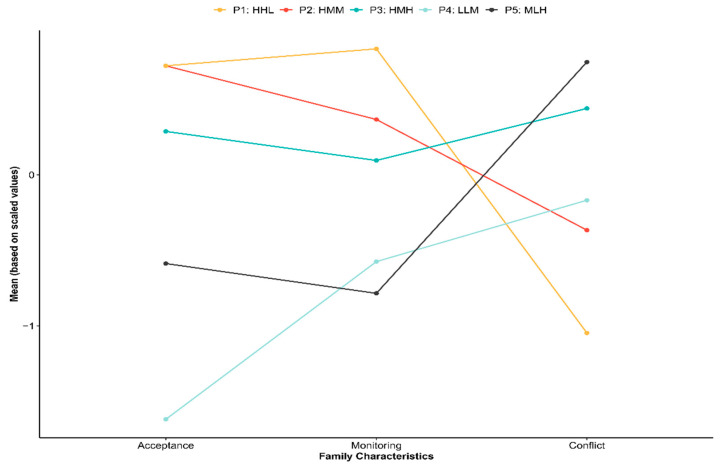
A visual depiction of the differences among the five family typologies. Mean of the standardized values are plotted. P1:HHL = *H-acceptance*, *H-monitoring*, *L-conflict*; P2:HMM = *H-acceptance*, *M-monitoring*, *M-conflict*; P3:HMH = *H-acceptance*, *M-monitoring*, *H-conflict*; P4:LLM = *L-acceptance*, *L-monitoring*, *M-conflict*; P5:MLH = *M-acceptance*, *L-monitoring*, *H-conflict*.

Latent profile models comprising profiles 1 through 5 were fit to the data. Model fit indices for each profile are shown in Table 1. The five-profile solution was selected as the best model as it had the lowest BIC, CAIC, and SABIC values, a significant BLRT *p*-value, and the highest entropy value. Means and standard deviations for the five-solution profile are displayed in Table 2. Profile names were selected based on means of each variable and defining differences between profiles. Consequently, families in our sample were labelled as: *high(H)-acceptance*, *H-monitoring*, *low(L)-conflict* (P1; *n* = 1465; 12%); *H-acceptance*, *medium(M)-monitoring*, *M-conflict* (P2; *n* = 3095, 26%); *H-acceptance*, *M-monitoring*, *H-conflict (*P3; *n* = 3357, 28%); *L-acceptance*, *L-monitoring*, *M-conflict (*P4; *n* = 1895, 16%); and *M-acceptance*, *L-monitoring*, *H-conflict* (P5; *n* = 2020, 17%). Figure 1 displays a plot of the scaled means for each of the five latent profiles.

### 3.2. Association of Family Profiles with Number of Movement Behaviors Met 

Table 3 shows the odds ratios for associations between family typologies and number of movement behaviors met. Results showed that children from families with *H-acceptance*, *M-monitoring*, *M-conflict* (P2; OR = 0.54); *H-acceptance*, *M-monitoring*, *H-conflict* (P3; OR = 0.28); *L-acceptance*, *L-monitoring*, *M-conflict* (P4; OR = 0.24); and *M-acceptance*, *L-monitoring*, *H-conflict* (P5; OR = 0.19) were at lower odds for meeting all three movement behaviors compared to children from families with *H-acceptance*, *H-monitoring*, *L-conflict* (P1; referent group). Similar results were found for the remaining recommendation categories; indeed, children from families with less-than-ideal functioning (P2–P5) were at lower odds for meeting ≥2 recommendations and ≥1 recommendation compared to children from families with *H-acceptance*, *H-monitoring*, *L-conflict*. Notably, the results showed that the odds of meeting a movement behavior category progressively decreased as family functioning worsened. For instance, the ORs for meeting ≥2 recommendations were 0.77, 0.49, 0.42, and 0.28 for children from families with *H-acceptance*, *M-monitoring*, *M-conflict* (P2); *H-acceptance*, *M-monitoring*, *H-conflict* (P3); *L-acceptance*, *L-monitoring*, *M-conflict* (P4); and *M-acceptance*, *L-monitoring*, *H-conflict* (P5), respectively. Furthermore, within a given family typology, as the number of recommendations met increased, the odds ratios decreased. For example, the odds of meeting ≥1 recommendation, ≥2 recommendations, and 3 recommendations for children from families with *M-acceptance*, *L-monitoring*, *H-conflict* (P5) were 0.54, 0.35, and 0.19, respectively.

## 4. Discussion

The purpose of this study was to identify typologies of families characterized by parental acceptance, parental monitoring, and family conflict, and to examine whether these family typologies were associated with the number of movement behavior recommendations met. Using LPA, we found that children from families in our sample could be meaningfully classified into one of five family typologies: *H-acceptance*, *H-monitoring*, *L-conflict* (P1); *H-acceptance*, *M-monitoring*, *M-conflict* (P2); *H-acceptance*, *M-monitoring*, *H-conflict* (P3); *L-acceptance*, *L-monitoring*, *M-conflict* (P4); and *M-acceptance*, *L-monitoring*, *H-conflict* (P5). Results from logistic regression analyses revealed that children from less-than-ideal functioning families (P2-P5) were at progressively lower odds of meeting all three movement behaviors compared to children from families with *H-acceptance*, *H-monitoring*, *L-conflict*. Results of our study also highlight that as the number of movement behavior recommendations increased, the odds of meeting each recommendation category (i.e., ≥1 recommendation, ≥2 recommendations, and 3 recommendations) progressively decreased within any given family typology. 

Overall, these findings generally align with previous research, demonstrating that families with certain qualities can either positively or negatively influence children’s health behaviors. Family qualities that have been linked with adverse health indicators among children include: family conflict; repeated episodes of anger and aggression; a lack of parental availability for, involvement in, and supervision of child activities; and relationships that are cold, unsupportive, and neglectful [33]. Furthermore, children from families with *M-acceptance*, *L-monitoring*, *H-conflict* had the lowest odds of meeting each recommendation category (i.e., ≥1 recommendation, ≥2 recommendations, and 3 recommendations). Families with high levels of conflict are often lacking in acceptance, warmth, supervision, and parental availability. These combined characteristics of a family have not only been associated with a wide range of mental (e.g., anxiety, depression) and physical (e.g., aches and pains) risks among children but also with various educational (e.g., poor academic performance) and social (e.g., risky behavior, drinking) outcomes [33]. Children from risky families are more likely than their peers to focus on tension reduction, distraction, and escape in stressful situations and fail to learn important self-regulatory skills. In contrast, children from healthy families experience a sense of emotional security and acquire behaviors that permit effective self-regulation. Thus, compared to their counterparts, the finding that children from families with *M-acceptance*, *L-monitoring*, *H-conflict* may experience greater difficulties accumulating physical activity, limiting screen time, and acquiring sufficient sleep is not surprising. Family interventions aimed at reducing conflict and increasing warmth and monitoring can help to promote the healthy development of the child.

Our findings should be interpreted while considering some limitations. The movement behaviors were measured using subjective assessments, which can increase measurement error and bias. More rigorous methods (e.g., objective assessments) are needed to further our understanding of the antecedents and outcomes of children’s movement behaviors. Finding ways to objectively measure screen time, in particular, should be a focus of future research. While examining total time spent on screens is important, information regarding time spent on specific platforms (e.g., Twitter, Instagram) would provide a more nuanced insight into the health impacts of screen media use among young people. Social desirability was not taken into account and therefore raises some concern regarding the presence of response biases. Furthermore, the relationship between family factors and children’s ability to meet the movement behavior recommendations is undoubtedly complex, and therefore, intervening variables (moderators and mediators) should be incorporated in future analytical models. Child temperament, which represents individual differences in reactivity and self-regulation [34], plays an important role in children’s social and psychological development. Considering child temperament when examining family typologies and the movement behaviors may help to identify individual differences among children that make them more or less likely to meet the guidelines.

Despite its limitations, our study has several strengths and makes important contributions to both the movement behavior and family health literature. First, a methodological strength of our study was the use of LPA; classifying individuals, rather than variables, into profiles revealed that families in our sample varied on acceptance, monitoring, and conflict. Second, this study, to our knowledge, is the first to examine family typologies and their relationship with the 24-h movement behaviors, and thus improves upon and extends current knowledge on this topic. Findings from this study could be used to help inform future family-targeted interventions aiming to improve movement behavior adherence in children. Third, the family typologies generated in our study (via LPA) can be used in future studies to determine whether similar homogenous profiles emerge across more diverse samples. If similar profiles are identified, a theoretically meaningful taxonomy of family typologies could be developed.

That children from families with *H-acceptance*, *H-monitoring*, and *L-conflict* were more likely to meet all three movement behaviors than children from less-than-ideal functioning families should be of interest to health researchers and practitioners. Parents/caregivers should focus on instilling healthy habits in children in the early years of their lives as this may be harder as children age. Some emerging research suggests that parents of children (6–13 years) may be hesitant to impose rules restricting children’s screen time because it could potentially lead to more conflict between the dyad as well as between siblings [35,36]. Parents have also expressed that curtailing children’s screen time would require significant energy as they would be responsible for finding and creating alternative activities for their children [36]. Some parents have admitted that their children’s digital media use makes their lives a bit easier by keeping their children occupied, allowing parents to do other activities (e.g., household chores, work-related tasks) [36]. Therefore, exposing children to different non-screen-based activities at a young age might help to reduce parental concerns and consequences related to implementing household screen time rules.

Another important implication of our study is that, coupled with high acceptance and low conflict, high parental monitoring was favorably related to children’s physical activity, screen time use, and sleep duration. This should not be confused with the notion of “helicopter parenting”, which is a term used to describe parents who are potentially over-involved in the lives of their child and who micromanage their child’s life by being overly protective and unwilling to let go [37], which is inherently different than knowing your child’s whereabouts and activities (i.e., parental monitoring). Parents should be reminded of this difference and aim to strike a balance when supervising their child’s whereabouts. The notion that excessive supervision can develop into helicopter parenting has been supported by empirical research whereby higher parental supervision was associated with higher perceived helicopter parenting [38].

## 5. Conclusions

Many previous studies on the role of the family on children’s physical activity, screen time, and sleep have examined family characteristics individually (i.e., variable-centered approaches). Our study extends previous research by using a person-centered approach to show how key family characteristics (acceptance, monitoring, and conflict) cluster together to generate homogeneous family typologies. To our knowledge, our study is the first to examine the associations between family typologies and movement behavior recommendations among children. Results showed that children in our sample were from one of five family typologies. Children from less-than-ideal functioning families (i.e., P2–P5) were less likely to meet all three movement behavior recommendations compared to children from families with *H-acceptance*, *H-monitoring*, and *L-conflict*. Additionally, results showed that the odds of meeting the movement behavior recommendation categories decreased as the number of recommendations met increased. These findings highlight the importance of the family environment when promoting healthy movement behaviors among children.

## Figures and Tables

**Table 1:** Model fit indices from latent profile analyses.

Solutions	BIC	CAIC	SABIC	BLRT *p*-Value	Entropy
1-Profile	97,839.01	97848.01	97810.41	-	1.00
2-Profile	88,667.48	88684.48	88613.46	0.001	0.745
3-Profile	84,198.61	84223.61	84119.16	0.001	0.820
4-Profile	81,853.51	81886.51	81748.64	0.001	0.737
5-Profile	79,035.78	79076.78	78905.48	0.001	0.843

Note. BIC = Bayesian Information Criterion; CAIC = Consistent Akaike’s Information Criterion; SABIC = Sample-Adjusted Bayesian Information Criterion; BLRT = Bootstrap Likelihood Ratio Test.

**Table 2:** Descriptive statistics for parental acceptance, parental monitoring, and family conflict for the 5-profile solution.

Profile	Acceptance		Monitoring		Conflict	
	M	SD	M	SD	M	SD
P1: H-Acceptance, H-Monitoring, L-Conflict (*n* = 1462)	15.00	0.00	24.07	0.80	0.00	0.00
P2: H-Acceptance, M-Monitoring, M-Conflict (*n* = 3095)	15.00	0.00	22.87	1.67	1.33	0.87
P3: H-Acceptance, M-Monitoring, H-Conflict (*n* = 3357)	14.34	0.47	22.17	1.93	2.90	2.07
P4: L-Acceptance, L-Monitoring, M-Conflict (*n* = 1895)	11.45	1.62	20.44	2.98	1.72	1.73
P5: M-Acceptance, L-Monitoring, H-Conflict (*n* = 2020)	13.02	1.10	19.89	2.98	3.50	2.01

Note. Ranges (minimum and maximum) for acceptance, monitoring, and conflict were 9–15, 5–25, and 0–9, respectively. H = high, M = medium, L = low.

**Table 3:** Associations between predictors and number of movement behavior recommendations met.

	≥1 Recommendation			≥2 Recommendations			3 Recommendations		
Predictors	OR	95% CI	*p*	OR	95% CI	*p*	OR	95% CI	*p*
(Intercept)	70.73	30.20, 165.67	<0.001	40.96	13.58, 123.51	<0.001	1.58	0.18, 13.76	0.677
Age (decimal years)	0.71	0.66, 0.77	<0.001	0.63	0.57, 0.69	<0.001	0.66	0.55, 0.79	<0.001
Sex (ref: girls, n = 5142)	1.20	1.10, 1.31	<0.001	1.43	1.27, 1.61	<0.001	1.18	0.94, 1.49	0.148
Ethnicity: African Americans ^a^ (n = 1503)	0.27	0.18, 0.41	<0.001	0.17	0.10, 0.27	<0.001	0.19	0.06, 0.58	0.004
Ethnicity: Caucasians ^a^ (n = 5861)	0.65	0.44, 0.96	0.032	0.72	0.46, 1.12	0.145	1.40	0.55, 3.55	0.477
Ethnicity: Hispanics ^a^ (n = 1765)	0.36	0.24, 0.54	<0.001	0.31	0.20, 0.50	<0.001	0.29	0.10, 0.79	0.015
Ethnicity: Multiracial ^a^ (n = 1367)	0.43	0.29, 0.64	<0.001	0.43	0.27, 0.69	<0.001	0.61	0.23, 1.61	0.318
Family income (1–10 scale, M = 7.24, SD = 2.42)	1.14	1.12, 1.16	<0.001	1.28	1.24, 1.32	<0.001	1.41	1.31, 1.53	<0.001
Family typology: P2 ^b^	0.81	0.69, 0.95	0.012	0.77	0.63, 0.94	0.012	0.54	0.39, 0.76	<0.001
Family typology: P3 ^b^	0.62	0.53, 0.73	<0.001	0.49	0.40, 0.59	<0.001	0.28	0.20, 0.40	<0.001
Family typology: P4 ^b^	0.54	0.45, 0.64	<0.001	0.42	0.33, 0.52	<0.001	0.24	0.16, 0.36	<0.001
Family typology: P5 ^b^	0.54	0.45, 0.64	<0.001	0.35	0.28, 0.44	<0.001	0.19	0.12, 0.29	<0.001

Note. P2 = high-acceptance, medium-monitoring, medium-conflict; P3 = high-acceptance, medium-monitoring, high-conflict; P4 = low-acceptance, low-monitoring, medium-conflict; P5 = medium-acceptance, low-monitoring, high-conflict; ^a^ reference group = Asians (n = 216); ^b^ reference group = P1 (high-acceptance, high-monitoring, low-conflict).

## Data Availability

The data that support the findings of this study are available from the ABCD study, but restrictions apply to the availability of these data, which were used under license for the current study and so are not publicly available. Data are however available from the authors upon reasonable request and with permission of the ABCD study.
